# Pregnancy in patients submitted to arterial embolization for uterine leiomyomas

**DOI:** 10.5935/1518-0557.20240071

**Published:** 2025

**Authors:** Gustavo Santos Rainato, Marina Antonini e Silva, Jessica Abreu Ferreira Vaz de Melo, Fernanda Jardim Gripp, Gabriela Santos Soares, Carlos Eduardo Diniz Couto, Ilveu Cosme Dias, Flávia Guimarães Rodrigues

**Affiliations:** 1 Medical Student at Faculdade Ciências Médicas de Minas Gerais (FCMMG), Belo Horizonte, Brazil; 2 Medical student at PUC-MG, Belo Horizonte, Brazil; 3 Universidade Federal de Minas Gerais (UFMG), Belo Horizonte, Brazil

**Keywords:** pregnancy, uterine artery embolization, uterine leiomyomas, fertility

## Abstract

**Objective:**

To determine the frequency of pregnancy in patients submitted to uterine artery embolization (UAE) for the treatment of uterine leiomyomas who undergo medical follow-up at a clinic in Belo Horizonte.

**Methods:**

This study consists of a retrospective cohort study. It is based on data analysis of electronic medical records of patients who underwent the embolization procedure for uterine leiomyomas between March 2003 and March 2018.

**Results:**

Out of a total of 587 patients who underwent the UAE procedure to treat leiomyomas, 150 expressed an interested in becoming pregnant. Among these patients, 88 successfully achieved pregnancy, representing a frequency of 58.7%. Among the symptoms and signs analyzed prior to the procedure, menorrhagia (97%) and dyspareunia (71%) were the most commonly observed in the analyzed patients. According to race/ethnicity, the majority (n=39) of the patients identified themselves as brown, and the intramural location (96%) was the most frequent.

**Conclusions:**

The study showed that 58.7% of the women who underwent UAE and expressed an interest in becoming pregnant were able to achieve pregnancy, with a 13.6% loss rate. However, further studies are needed to elucidate the key variables that contribute to the increased likelihood of pregnancy after leiomyoma embolization procedure, as well as the effectiveness of symptoms reduction and the actual improvement in pregnancy rates.

## INTRODUCTION

Uterine leiomyomas or myomas are benign tumors developed from smooth muscle cells in women of reproductive age (Florence & Fatehi, 2024). They are often encountered as part of the investigation of a couple experiencing infertility, and their origin is monoclonal (Salman & Davis, 2010; Whynott *et al.*, 2017). About 75% of cases of leiomyomas are asymptomatic and usually found unexpectedly on abdominal, bimanual pelvic examination or ultrasound (Corleta *et al.*, 2007). They are hormone-responsive and estrogen promotes their growth (Okolo, 2008). Increased uterine bleeding is the most common complaint of this tumor and can lead to anemia (Wegienka *et al.*, 2004).

The presence of asymptomatic uterine leiomyomas in women generally does not require treatment. Instead, it is recommended to monitor them through routine gynecological exams. On the other hand, symptomatic uterine leiomyomas should be treated on an individual basis, taking into consideration factors such as the size and location of this benign tumor, as well as the patient’s interest in preserving the uterus and fertility (Corleta *et al.*, 2007; Vilos *et al.*, 2015). Subserous leiomyomas tend to cause compressive symptoms and anatomical distortion of adjacent organs, intramurals cause bleeding and dysmenorrhea, while submucosal ones more frequently produce irregular bleeding (Flake *et al.*, 2003). Based on this assessment, surgical or pharmacological treatment or a treatment with both modalities may be recommended (Corleta *et al.*, 2007; Vilos *et al.*, 2015; Ludwig *et al.*, 2020).

Uterine artery embolization (UAE) has become a treatment option for symptomatic uterine leiomyomas (Vilos *et al.*, 2015; Keung *et al.*, 2018). Its use has expanded as a minimally invasive treatment modality, used in situations where the only alternative is hysterectomy and the patient want to get pregnant or to preserve the uterus (Gupta *et al.*, 2012). The objective of the procedure is to reduce the size of the leiomyoma by injecting polyvinyl alcohol particles or embospheres into the uterine arteries followed by catheterization, a study published in 2020 showed a 37.4% reduction in the size of tumors (Ludwig *et al.*, 2020; Silva *et al.*, 2020). Other procedures such as laparotomy or laparoscopic myomectomy are considered the gold standard for conservative treatment of leiomyomas, while hysterectomy is considered a radical treatment option (Vilos *et al.*, 2015).

The first case of the use of UAE was published by Ravina *et al.* (1995) for the treatment of leiomyoma in 16 patients. After 20 months of follow-up, 11 of 16 patients had resolution of symptoms, restoring normal menstrual cycles in 10 of 11 responders (Ravina *et al.*, 1995). The first pregnancy outcomes after UAE were reported in 2000 and 2001 (Ravina *et al.*, 2000; McLucas *et al.*, 2001). In a later study of 112 patients who underwent UAE for the treatment of uterine leiomyoma, nine desired conservative treatment to preserve reproductive capacity. Among these patients, four achieved pregnancy, two experienced early miscarriage, and two progressed normally until the end of pregnancy, resulting in full-term deliveries (Bonduki *et al.*, 2006). In a later study conducted in 2013, which analyzed the evolution and delivery outcomes after UAE, it was found that out of 98 patients undergoing treatment for symptomatic leiomyomas, 21 expressed interest in getting pregnant, of whom seven successfully achieved pregnancy (Redecha *et al.*, 2013).

Manifold scientific studies point to UAE as a safe and effective procedure for the treatment of uterine leiomyomas, and has similar complications as myomectomy (Schirf *et al.*, 2006; Kohi & Spies, 2018; Ludwig *et al.*, 2020; Manyonda *et al.*, 2020; Cornman-Homonoff *et al.*, 2021). However, the authors reinforce the need for further studies on the effects of this procedure on fertility and future pregnancy. Thus, the objective of the present study was to evaluate the proportion of women submitted to UAE that becomes interested in getting pregnant and to evaluate the epidemiological and clinical data, pregnancy rate and reproductive outcomes of these women who undergo medical follow-up at a gynecological clinic in Belo Horizonte, Minas Gerais, Brazil.

## MATERIAL AND METHODS

### Study design

The study was conducted as a retrospective cohort research based on information obtained from the electronic database of a clinic in Belo Horizonte, Minas Gerais State, regarding patients submitted to arterial embolization for the treatment of uterine leiomyomas. The present study was submitted and approved by the Research Ethics Committee of Ciências Médicas de Minas Gerais (CEPCM-MG), with a Certificate of Presentation for Ethical Appreciation (CAAE: 52643221.0.0000.5134).

### Sample

The study analyzed data from 150 patients who underwent arterial embolization for the treatment of uterine leiomyomas out of a total of 587 procedures performed between March 2003 and March 2018 in a gynecological clinic. However, the data collected and analyzed were obtained specifically from 88 patients who achieved pregnancy after the arterial embolization procedure for treating leiomyomas.

### Clinical follow-up

The standard follow-up after the UAE procedure consisted of three appointments with the gynecologist at 3-, 6- and 12-months post-procedure. For patients aged 35 years or younger who wish to conceive after the 12-months period, referral to other therapies, such as artificial insemination, in vitro fertilization or ovulation induction, is recommended to enhance pregnancy rates. Patients with more than 35 years or older are referred to these other therapies with six months after the procedure. In both cases, patients referred to other therapies were removed from the study. Patients who achieved pregnancy without any additional therapy within one year of the procedure were added in the study based on spontaneous appointments at the clinic.

### Inclusion and exclusion criteria

The study included data from electronic medical records of patients submitted to arterial embolization for the treatment of uterine leiomyomas aged over 20 years and less than or equal 40 years who were interested in getting pregnant. It was also included patients who were not referred to other therapies and had a confirmed diagnosis of leiomyoma.

### Epidemiological, clinical and radiological data

For the development of the study, epidemiological data such as age, race/ethnicity and origin, as well as clinical manifestations and radiological findings, were collected from the patients who underwent the procedure. The following clinical parameters were collected and analyzed: presence or absence of menorrhagia, dyspareunia, severe cramps, urinary dysfunction, time between diagnosis of leiomyomas and confirmation of pregnancy (laboratory test: positive β-HCG). Regarding the radiological parameters, the number and location of leiomyomas (submucosal, subserous and intramural) were observed.

### Data analysis

After data collection, these were compiled and tabulated in the Microsoft Excel spreadsheet editor. The categorical variables were presented as absolute and relative frequencies and the numeric variables as average ± standard deviation and median (1st quartile - 3rd quartile). The associations of categorical variables with the classification were evaluated by the Chi-square test and the comparisons of numerical variables with the classification by the Kruskal-Wallis test with multiple comparisons using the Nemenyi test. The analyzes were performed using the R software version 4.0.3 considering a significance level of 5%.

## RESULTS

In the study, a total of 587 patients submitted to UAE for the treatment of leiomyomas, 150 wanted to get pregnant. Of these, 88 were successful, which constitutes a pregnancy rate of 58.7%. Furthermore, of these 88 patients who were successful in getting pregnant, 76 patients had children born alive, corresponding to a frequency of 86.4% and 13.6% had miscarriages (n=12).

According to the analysis of the epidemiological profile of the patients who underwent UAE and became pregnant, it was observed that the most frequent age group was between 30 and 40 years old, corresponding to 89.8% (n=79) of the patients analyzed. According to the informed race/ethnicity, 44.3% (n=39) of the patients identified themselves as brown, 28.4% (n=25) black, 21.6% (n=19) white and 5, 7% (n=5) did not inform. Of the analyzed population who were followed up at the gynecological clinic, 94.3% of the patients came from Minas Gerais and 5.7% from other Brazilian states (Table 1). The age median is 30.45, the first quartile 30.17, the third one is 30.74, making the interquartile range 0.57 years.

**Table 1:** Main characteristics of patients undergoing treatment for uterine leiomyomas who became pregnant after arterial embolization.

Characteristics	Absolute frequency	Relative frequency (%)
Age 20-29 30-40	09 79	10.2% 89.8%
Race/Ethnicity White Black Brown Unevaluated	19 25 39 05	21.6% 28.4% 44.3% 5.7%
Origin Minas Gerais Others	83 05	94.3% 5.7%
Signs and symptoms Menorrhagia Presence of intense cramps Urinary dysfunction Dyspareunia	85 10 16 63	97% 11% 18% 71%

Among the signs presented by the patients before UAE, the most frequent were menorrhagia with 97% (n=85) and dyspareunia with 71% (n=63). Urinary dysfunction with 18% (n=16) and intense cramps with 11% (n=10) were the least frequent signs and symptoms, according to Table 1.

Regarding the time between the arterial embolization procedure for treatment of uterine leiomyomas and the occurrence of pregnancy, 69.3% (n=61) of women had a positive diagnosis between 7 and 11 months and 12.5% (n=11) between 12 and 16 months. The frequency of women who were successful in becoming pregnant 17 months after UAE was 4.5% (n=4) (Table 2). Thus, the mean value between the time of performing UAE and the occurrence of pregnancy was 9.83 months (SD=4.59 months) with a variability of approximately 46.7%. The first quartile found for the time variable was 8 months with a third quartile equal to 11 months.

**Table 2:** The mean time in months between treatment for uterine leiomyomas and the occurrence of pregnancy was determined for patients who underwent follow-up at a clinic in Belo Horizonte, Minas Gerais.

Months	Absolute frequency	Relative frequency (%)
Under 6	8	9.1%
Between 7 e 11	61	69.3%
Between 12 e 16	11	12.5%
Between 17 e 21	1	1.1%
Between 22 e 26	2	2.3%
Between 27 e 31	0	0.0%
Above 32	1	1.1%
Unevaluated	4	4.5%
Total	88	100%

When examining the association between the number of leiomyomas, race/ethnicity, the signs and symptoms observed and the average time between UAE and the pregnancy diagnosis, a statistically significant difference was found only in relation to the symptom of dyspareunia (*p*=0.039). In this comparison, women who had less than five leiomyomas had this complaint in 62% of the cases (n=28), those who had between five and nine complained of dyspareunia in 86% (n=30), and of those who had more than nine, 62% had the symptom (n=5). Furthermore, comparing the number of leiomyomas with the average time between UAE and pregnancy (*p*=0.04) it was observed that 81% (n=34) of the women who had less than five and 59% (n=20) between five and nine leiomyomas became pregnant with less than 11 months. Among the women with less than five leiomyomas, 19% (n=8) became pregnant later. While 41% (n=14) of those with between five and nine leiomyomas achieved pregnancy. In the group of women with more than nine leiomyomas, 50% (n=4) received a pregnancy diagnosis within 11 months, while the remaining 50% (n=4) received the diagnosis after the analyzed time interval (Table 3).

**Table 3:** Association between the number of leiomyomas, race/ethnicity, signs and symptoms and the average time to pregnancy in months.

Characteristics	N	Under 5 n=45	Between 5 and 9 n=35	Superior to 9 n=8	*p* value
**Race/ethnicity** White Black Brown	83	7 (18%) 13 (32%) 20 (50%)	8 (23%) 10 (29%) 17 (49%)	4 (50%) 2 (25%) 2 (25%)	0.4
**Signs and symptoms** Menorrhagia Dyspareunia Presence of cramps Urinary disfunction	85 63 10 16	42 (93%) 28 (62%) 4 (8.9%) 8 (18%)	5 35 (100%) 30 (86%) (14%) (17%)	1 8 (100%) 5 (62%) (12%) (25%)	0.4 **0.039** 0.6 0.8
**Time between the arterial embolization and pregnancy** Less than 11 months More than 11 months	84	34 (81%) 8 (19%)	20 (59%) 14 (41%)	4 (50%) 4 (50%)	**0.040**

## DISCUSSION

Uterine artery embolization (UAE) has been utilized as a minimally invasive procedure for the treatment of uterine leiomyomas. It serves as a therapeutic alternative for women who desire to preserve their reproductive capacity, particularly when the leiomyoma is associated with infertility and is unresponsive to other conservative surgical treatments, or when previous conservative treatment has been unsuccessful (Pron *et al.*, 2005). In the proposed study the frequency of pregnancy in patients undergoing UAE in a gynecological clinic in Belo Horizonte, Minas Gerais was demonstrated.

In the proposed study, a 58.7% frequency of pregnancy was observed in patients undergoing the UAE procedure for the treatment of uterine leiomyomas. A study in 2013 observed that of 98 patients undergoing UAE, 21 expressed interest in becoming pregnant, of these seven became pregnant, showing a pregnancy frequency of 33.3% (Redecha *et al.*, 2013). Later, in the study developed involving 359 patients undergoing UAE, a frequency of 41.5% reproductive success was observed, that is, 149 of the 359 patients who underwent the procedure were able to become pregnant. Furthermore, the study describes 87.9% success rate in these pregnancies, 131 of 149 women had pregnancies with live-born children (Pisco *et al.*, 2017).

In the study developed by Pisco *et al.* (2017), who evaluated the epidemiological profile of patients undergoing UAE, it was observed that the cumulative probability of successful pregnancy at 24 months was 32.6% for women aged 40 years or older and 35.7% for women younger than 40 years (*p*=0.67). When analyzing the epidemiological profile of patients undergoing the procedure in the current study, it was observed that there was a predominance of 30 to 40 years of age (89.8%) and most of them belonging to the mixed race/ethnicity (44.3%) (Pisco *et al.*, 2017).

Earlier studies have shown that patients with uterine leiomyomas have reduced pregnancy and embryo implantation rates (Torre *et al.*, 2014; Pisco *et al.*, 2017; Krzyzanowski *et al.*, 2022). However, the location of these benign neoplasms can affect reproductive success (Carranza-Mamane *et al.*, 2015; Sundermann *et al.*, 2017). According to a relevant number of scientific studies, there is no association between the presence of leiomyomas of subserosal location with infertility (Pritts *et al.*, 2009; Carranza-Mamane *et al.*, 2015; Manyonda *et al.*, 2020). On the other hand, it is not completely elucidated whether the presence of intramural leiomyomas reduces fertility (Carranza-Mamane *et al.*, 2015; Sundermann *et al.*, 2017). In the current study, only women with intramural or subserous uterine leiomyomas who underwent arterial embolization were able to achieve pregnancy.

In the study proposed by Keung *et al.* (2018), it was observed that the location of leiomyomas results in different clinical repercussions, and cites that the presence of menorrhagia, dysmenorrhea, uterine cavity enlargement, and infertility are the most frequently observed symptoms in patients with this pathological change. The current study analyzed four main symptoms, the most frequent being: menorrhagia, present in 97% (n=85), followed by dyspareunia and presence of intense cramps, respectively, corroborating with the other study developed by Kim *et al.* (2018). Moreover, in the article by Kim *et al.* (2018) the most prevalent symptom was menorrhagia with 87.3%, which reinforces the findings of the current study.

The mean time from UAE to pregnancy diagnosis in the proposed study was 9.8 months, with a standard deviation of 4.59 months and a variability of 46.7%. The study identified a potentially influential variable that may explain the time discrepancy, which was the number of leiomyomas (*p*=0.04). Women with up to five leiomyomas (81%) exhibited a higher likelihood of achieving reproductive success within 11 months following the procedure. In contrast, women who had between five and nine leiomyomas (59%) or more than nine (50%) took more than 11 months to be diagnosed with pregnancy. According to Pisco *et al.* (2017), the average time between leiomyoma treatment and diagnosis of first pregnancy in patients undergoing UAE was 12 months, similar to that observed in the proposed study.

Previous studies have shown significant differences in the outcomes, and one important contributing factor is the novelty of the UAE procedure. Therefore, the success rate among different surgeons is a relevant factor that can explain the variations in the results. Another important aspect is the considerable variability in the number of patients on each study, which can lead to variations in outcomes. Therefore, the inclusion of a sufficient number of patients in this current study is crucial for contributing to the existing literature.

Since the clinical follow up after 12 months from the procedure was spontaneous, this might be considered a limitation. Due to some of the patients that were pregnant after 12 months might not come back to a doctor’s appointment spontaneously, the average time between the procedure and the pregnancy data may be underestimated. Furthermore, since this is an observational study without control groups, it becomes challenging to assess the true efficacy of the UAE procedure in reducing symptoms and improving pregnancy rates. This limitation reduces the external validation of the study. Nevertheless, the findings of this study are significant for assessing the average time between the procedure and pregnancy, as well as for analyzing the epidemiological and clinical data of patients who successfully achieved pregnancy. Our data will contribute with the literature and might be important to novel studies on leiomyomas treatments.

## CONCLUSION

The study demonstrated that 58.7% of the women underwent UAE and desired pregnancy, were able to achieve it, with a 13.6% loss rate. However, further studies are needed to elucidate which variables are key in the increased likelihood of pregnancy after leiomyoma embolization procedure and the effectiveness in reducing symptoms of this procedure and the real improvement in the pregnancy rates.
